# PKR Promotes Oxidative Stress and Apoptosis of Human Articular Chondrocytes by Causing Mitochondrial Dysfunction through p38 MAPK Activation—PKR Activation Causes Apoptosis in Human Chondrocytes

**DOI:** 10.3390/antiox8090370

**Published:** 2019-09-03

**Authors:** Ching-Hou Ma, Chin-Hsien Wu, I-Ming Jou, Yuan-Kun Tu, Ching-Hsia Hung, Wan-Ching Chou, Yun-Ching Chang, Pei-Ling Hsieh, Kun-Ling Tsai

**Affiliations:** 1 Department of Orthopedics, E-Da Hospital, I-Shou University, Kaohsiung City 82445, Taiwan; 2 School of Medicine, College of Medicine, I-Shou University, Kaohsiung City 82445, Taiwan; 3 Department of Physical Therapy, College of Medicine, National Cheng Kung University, Tainan 701, Taiwan; 4 Institute of Allied Health Sciences, College of Medicine, National Cheng Kung University, Tainan 701, Taiwan; 5 Department of Nursing, Shu-Zen Junior College of Medicine and Management, Kaohsiung City 82445, Taiwan; 6 Department of Anatomy, School of Medicine, School of Medicine, China Medical University, Taichung 404, Taiwan

**Keywords:** osteoarthritis, chondrocyte, PKR, oxidative stress, mitochondrial dysfunction

## Abstract

Osteoarthritis (OA) is one of the most common types of arthritis in the elderly people. It has been known that chondrocyte apoptosis occurs in OA cartilage; however, the detailed molecular mechanism remains unclear. In the current study, we aimed to elucidate the role of double-stranded RNA-dependent protein kinase R (PKR) in the TNF-α-caused apoptosis in chondrocytes. Human articular chondrocytes were digested from cartilages of OA subjects who accepted arthroplastic knee surgery. Our results showed that phosphorylation of p38 MAPK was increased after TNF-α stimulation or PKR activation using poly (I:C), and TNF-α-induced p38 MAPK upregulation was inhibited by PKR inhibition, suggesting phosphor-p38 MAPK was regulated by PKR. Moreover, we found that PKR participated in the p53-dependent destruction of AKT following activation of p38 MAPK. The inhibition of AKT led to the reduced expression of PGC-1α, which resulted in mitochondrial dysfunction and increased oxidative stress. We showed that the reduction of oxidative stress using antioxidant Mito TEMPO lowered the TNF-α-induced caspase-3 activation and TUNEL-positive apoptotic cells. The diminished apoptotic response was also observed after repression of PKR/p38 MAPK/p53/AKT/PGC-1α signaling. Taken together, we demonstrated that the aberrant mitochondrial biogenesis and increased oxidative stress in chondrocytes after TNF-α stimulation were mediated by PKR, which may contribute to the chondrocyte apoptosis and cartilage degeneration in OA.

## 1. Introduction

Osteoarthritis (OA) has been recognized as a degenerative joint disease affecting the articular cartilage and its surrounding tissues. Osteochondral changes occur during the development of OA, including a breakdown of articular cartilage, remodeling of the underlying bone, inflammation of the synovium as well as hypertrophy of the joint capsule [1]. As the most prevalent joint disease and a leading cause of chronic pain and disability, OA causes a great burden to the aging population since the prevalence increases with age, especially in adults age ≥50 years [2]. Aside from a decline in physical function [3], OA also has detrimental psychological effects [4]. Moreover, it has been suggested that knee OA has doubled in prevalence since the mid-20th century [5]. As such, it is imperative to better understand the pathogenesis of OA in order to delay or prevent the cartilage degeneration.

Current knowledge has shown that OA is associated with the breakdown of joint tissues due to mechanical loading [6] and chronic inflammation [1]. Indeed, various studies have revealed that matrix metalloproteinases (MMP) regulatory network was induced by inflammation [7]. Moreover, chondrocyte apoptosis following inflammation also contributes to the subsequent development of OA as normal articular cartilage homeostasis and structural integrity depend on human chondrocytes [8,9,10]. Chondrocyte apoptosis involves a complex process mediated by numerous intercellular signaling pathways, such as IL-1β-associated nitric oxide production [11,12]. Another overproduced inflammatory mediator, TNF-α, is capable of inducing apoptosis in articular chondrocytes as well [13]. It has been shown that TNF-α and IL-1β control apoptosis differently in human chondrocyte [14,15]. For instance, the TNF-α-increased caspase-1 and -8 mRNA levels were not observed in the IL-1β treatment group [14]. Nevertheless, the exact molecular mechanism underlying the TNF-α-induced chondrocyte apoptosis still requires more effort to unveil.

The double-stranded RNA-dependent protein kinase R (PKR) is a serine/threonine kinase that extensively expressed in mammalian cells. PKR is one interferon-inducible kinase. The up-regulation of PKR leads to phosphorylation of its physiological substrate, the α-subunit of eukaryotic initiation factor 2 (eIF-2α). In OA chondrocytes, the elevated phosphorylation of PKR was reported [16]. It has been shown that inhibition of PKR antagonized the IL-1-activated eIF2α phosphorylation, leading to reduced proteoglycan degradation and cyclooxygenase (COX)-2 accumulation [17]. It has also been found that the enhanced MMPs secretion in OA chondrocytes was due to the increased phosphorylation of PKR [16,18]. Moreover, accumulating evidence has suggested that TNFα-induced apoptosis requires a functional PKR pathway [19,20]. Since the increased PKR phosphorylation was shown in OA chondrocytes and PKR is pivotal in TNF-α-induced apoptosis, we sought to investigate whether PKR participates in TNF-α-induced chondrocyte apoptosis. As a result, we conducted a series of experiments to examine this hypothesis and decipher the associated molecular signaling pathway.

## 2. Materials and Methods

### 2.1. Reagents

Culture medium Dulbecco’s modified Eagle’s medium (DMEM) and Trypsin-EDTA were purchased from Gibco (Grand island, NY, USA). Polyinosinic-polycytidylic acid (poly(I:C)), SB203580, SC 79, ZLN005, C16, mito TEMPO, Pifithrin-μ, streptomycin and penicillin were all purchased from Sigma (St. Louis, MO, USA). The concentrations of poly(I:C), penicillin, and streptomycin were referred from our previous study [16,21]. The concentration of SB203580 was referred from our previous report [22]. The concentration of SC 79 was selected by our preliminary tests (data not shown). The concentrations of ZLN005, C16, Pifithrin-μ and mito TEMPO were referred from published studies [23,24,25,26]. Anti-p38, anti-p-p38, anti-p53, anti-p-p53, anti-AKT, anti-p-AKT, anti-PGC-1α, and anti-β-actin were all bough from Cell Signaling Technology (Danvers, MA, USA). Secondary antibodies and recombinant TNF-α protein were obtained from Abcam Inc. (Cambridge, MA, USA). MitoSOX and JC-1 were obtained from Thermo Scientific (IL, USA). Antioxidant superoxidase dismutase SOD kit and ApopTag^®^ Peroxidase In Situ Apoptosis Detection Kit were obtained from EMD Millipore (Gibbstown, NJ, USA).

### 2.2. Chondrocytes Isolation

The protocol of this study was approved by the Ethics Committee of E-Da Hospital (EMRP-105-077), and each participant provided signed informed consent. The articular cartilages of knee joint were collected from OA cases that accepted arthroplastic knee surgery. The articular cartilages were digested for in vitro investigations. Cartilage tissues were cut into small pieces and washed with Phosphate buffered saline (PBS). The collagenase B plus DMEM was used to digest articular cartilages under 16 h incubation. The isolated chondrocytes were collected by centrifugation (1500 rpm/5 min). Chondrocytes were cultured in Dulbecco’s Modified Eagle Medium (DMEM) with 10% FBS, 2 mM l-glutamine, 25 mM 4-(2-hydroxyethyl)-1-piperazineethanesulfonic acid (HEPES), 100 U/mL penicillin, and 100 mg/mL streptomycin at 37 °C CO_2_ incubator [27].

### 2.3. Investigation of Proteins

Radioimmunoprecipitation assay buffer (RIPA) buffer was bought to extract lysate. The proteins were transferred on to a polyvinylidene difluoride membrane after the proteins were separated by SDS/PAGE. After blocking with 5% nonfat milk in PBST (PBS with 0.1% Tween 20) for 1 h, the membrane was washed by PBST and incubated with primary antibodies overnight at 4 °C. After incubation of primary antibodies, membranes were incubated with HRP-conjugated secondary antibody for 1 h. Membranes were washed and detected with the Enhanced chemiluminescence (ECL) system (Millipore).

### 2.4. Measurement of Mitochondria ROS Concentration

Reactive oxygen species (ROS) concentrations were measured by mitoSOX. Confluent cells (10^4^ cells/well) in 96-well plates were treated with TNF-α for 24 h in treatment group. After TNF-α treatment, mitoSOX (10 μM) was loaded for 1 h incubation. Fluorescence intensity was measured with a flow cytometry.

### 2.5. PKR Knockdown

ON-TARGET plus SMART pool small-interfering RNA (siRNA) for si-Control was bought from Dharmacon Research. si-PKR was bought from Santa Cruz. Transfections of si-PKR and si-Control were performed using INTERFERin siRNA transfection kit (Polyplus Transfection, Huntingdon, UK) according to the protocol.

### 2.6. Investigation of Mitochondrial Membrane Potential

The JC-1 is widely used to study mitochondrial membrane potential. In health cells, JC-1 concentrates in the mitochondrial matrix where it forms red fluorescent aggregates. In apoptotic and necrotic cells, JC-1exists in monomeric form and stains cells green. After stimulation of TNF-α for 24 h, cells were rinsed with DMEM and then loaded with JC-1 (5 μM). After a 30-min incubation at RT, cells were assayed by flow cytometry.

### 2.7. Mitochondrial Biogenesis

The N-nonyl acridine orange (NAO) staining was used examining mitochondrial mass. Chondrocytes were treated with TNF-α and incubated with 5 µM NAO for 30 min at 37 °C, and cells were assayed by flow cytometry. Real-time PCR assay was performed to investigate mitochondrial DNA (mtDNA) content. The primers of mitochondrial complex II: Sense primer 5′-CAAACCTACGCCAAAATCCA-3′ and antisense primer 5′-GAAATGAATGAGCCTACAGA-3′ and β-actin: Sense primer 5′-AGGTCATCACTATTGGCAACGA-3′ and antisense primer 5′-CACTTCATGATGGAATTGAATGTAGTT-3′. PCR was assayed by SYBR Green on an ABI 7000 sequence detection system according to the protocol.

### 2.8. Antioxidant Activity

SOD activity in chondrocytes was studied via an enzymatic assay using a commercial kit (Calbiochem, 574601) according to the protocol.

### 2.9. Investigation of Apoptosis

Apoptotic cells were analyzed by the ApopTag^®^ Peroxidase In Situ Apoptosis Detection Kit (Calbiochem). After treatment with TNF-α for 24 h, cells were rinsed twice in PBS before fixation for 30 min at room temperature with 4% paraformaldehyde. Next, cells were washed in PBS before incubation in the prepared solution (0.1% Triton X-100, 0.1% sodium citrate) for 5 min. Cells were then incubated with 1 TUNEL reaction mixture in a humidified atmosphere for 1 h at 37 °C in the dark, washed in PBS, and analyzed by flow cytometry. The BioVision CaspGLOW™ Fluorescein Active Caspase-3 Staining Kit (Milpitas, CA, USA) was used for detection of active caspase 3.

### 2.10. Statistical Analyses

The results are expressed as mean ± SD. Statistical analyses were performed using a one-way or two-way ANOVA, followed by a Tukey’s test as appropriate. A *p*-value < 0.05 was considered statistically significant.

## 3. Results

### 3.1. TNF-α Activates p38 MAPK via PKR in Chondrocytes

**Figure 1:**
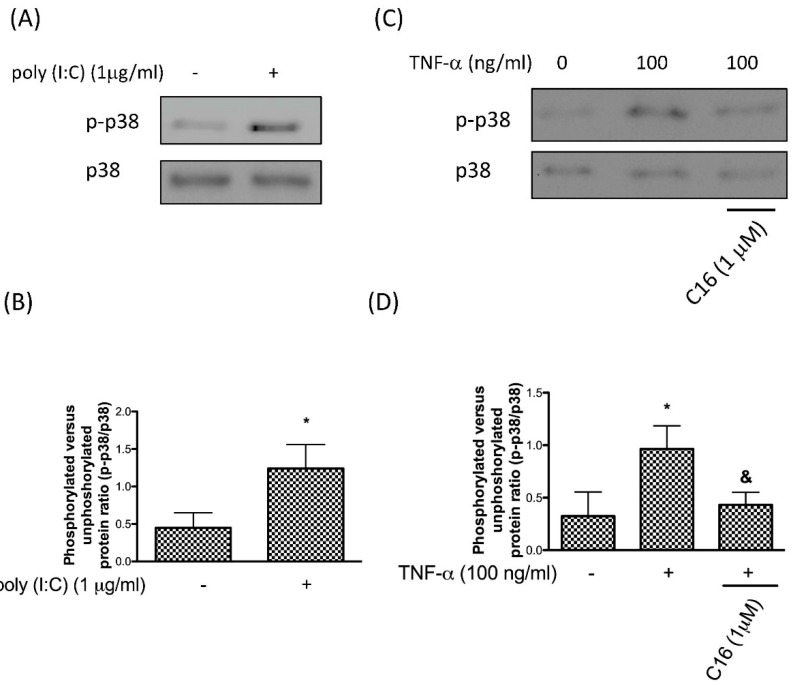
Protein expression (**A**) and the ratio (**B**) of p-p38 after upregulation of PKR using poly (I:C); protein expression (**C**) and the ratio (**D**) of p-p38 after exogenous administration of TNF-α for 12 h in human chondrocytes with or without PKR inhibitor C16 (*n* = 3; * *p* < 0.05 compared to control group. *p* < 0.05 compared to TNF-α group, *p* values were generated by ANOVA using the Tukey’s test).

It has been known that TNF-α induces p38 MAPK activation during the inflammatory response at the injured sites [28], and this induction is important for TNF-α-mediated bone destruction in arthritis [29]. Given that PKR is required for p38 MAPK activation [30], it is tempting to speculate that PKR participates in the TNF-α-induced p38 MAPK activity. First of all, we treated cells with a synthetic analog of dsRNA polyinosinic-polycytidylic acid (poly (I:C)) for 24 h to enhance the activity of PKR. This time point and dosage were referred to our previous study [16]. Results showed that the increased PKR expression in chondrocytes resulted in an elevation of phosphor-p38 MAPK (Figure 1A,B). In our previous work, we have demonstrated that TNF-α upregulated the activity of phosphor-PKR in chondrocytes [16]. As shown in Figure 1C,D, the TNF-α-induced p38 MAPK upregulation was abrogated by PKR inhibitor C16, suggesting that PKR was required for the increase in phosphor-p38 MAPK following TNF-α treatment.

### 3.2. Phosphorylation of p53 after TNF-α-Induced p38 MAPK Activation Is Mediated by PKR

**Figure 2:**
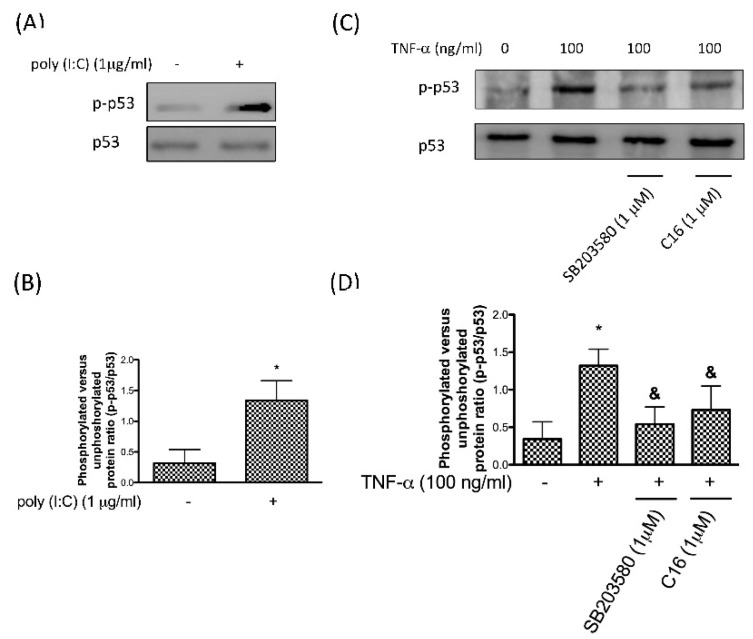
Protein expression (**A**) and the ratio (**B**) of p-p53 after upregulation of PKR using poly (I:C); Protein expression (**C**) and the ratio (**D**) of p-p53 following TNF-α exposure in human chondrocytes in the presence or absence of p38 inhibitor SB203580 or PKR inhibitor C16 (*n* = 3; * *p* < 0.05 compared to control group. *p* < 0.05 compared to TNF-α group, *p* values were generated by ANOVA using the Tukey’s test).

Higher expression of p53 has been found in the OA chondrocytes [31] and p38 MAPK-mediated p53 phosphorylation constitutes a critical step of apoptosis [32,33]. In order to examine whether PKR activation is involved in p53 phosphorylation, poly (I:C) was used. The use of poly (I:C) showed that PKR unregulated the levels of phosphor-p53 (Figure 2A,B). To reveal its upstream regulators, we showed that levels of phosphorylated of p53 were increased after TNF-α stimulation, while this activation was blocked in the presence of p38 inhibitor SB203580 or PKR inhibitor C16 (Figure 2C,D). Collectively, we demonstrated that phosphorylation of p53 in chondrocytes under inflammation may be regulated by the PKR/p38 MAPK pathway.

### 3.3. Downregulation of Phosphor-AKT by TNF-α Stimulation Is through the PKR/p38 MAPK/p53 Pathway

**Figure 3:**
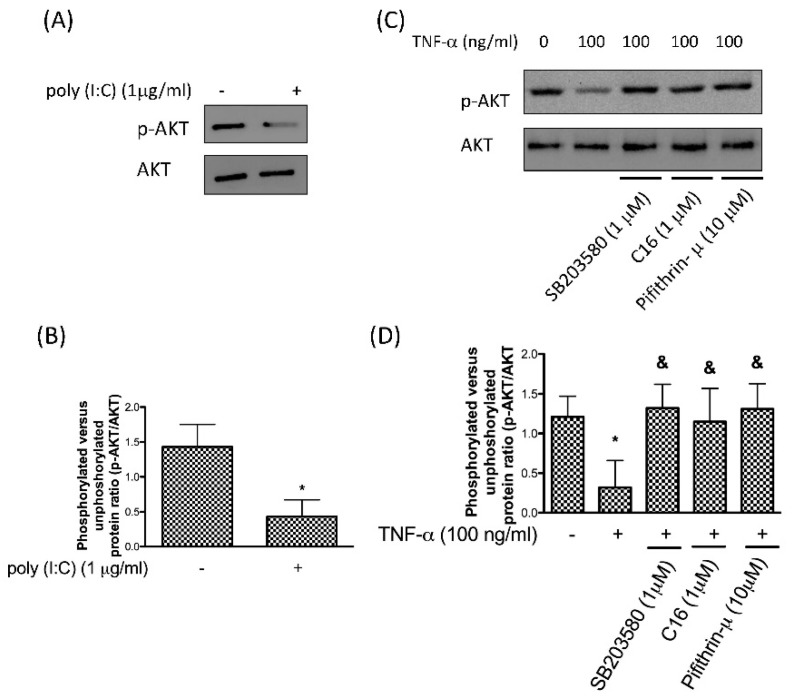
Protein expression (**A**) and the ratio (**B**) of p-AKT after activation of PKR using poly (I:C); protein expression (**C**) and the ratio (**D**) of p-p53 after stimulation of TNF-α in human chondrocytes in the presence or absence of p38 inhibitor SB203580, PKR inhibitor C16 or p53 inhibitor Pifithrin-μ. *n* = 3; * *p* < 0.05 compared to control group. *p* < 0.05 compared to TNF-α group, *p* values were generated by ANOVA using the Tukey’s test.

p53 and AKT play crucial roles in the transduction of pro-apoptotic and anti-apoptotic signals, respectively. It has been proven that p53-dependent downregulation of AKT promotes the commitment to apoptotic cell death [34]. To verify whether PKR participated in the p53-dependent destruction of AKT we treated chondrocytes with poly (I:C) and demonstrated that the expression of phosphor-AKT was diminished following upregulation of PKR (Figure 3A,B). Next, we showed that administration of TNF-α reduced the expression of phosphor-AKT whereas this downregulation was prevented by p38 inhibitor SB203580, PKR inhibitor C16, or a specific p53 inhibitor Pifithrin-μ (Figure 3C,D). Our findings imply that TNF-α-inhibited phosphorylation of AKT was mediated by the PKR/p38 MAPK/p53 pathway.

### 3.4. Reduction of PGC-1α by TNF-α Is via the PKR/p38 MAPK/p53/AKT Pathway

**Figure 4:**
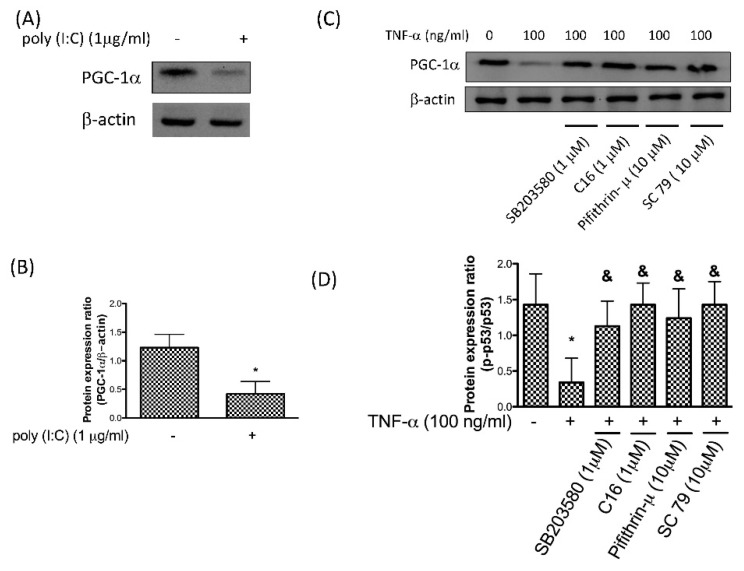
Protein expression (**A**) and ratio (**B**) of PGC-1α after activation of PKR using poly (I:C); protein expression (**C**) and ratio (**D**) of PGC-1α after TNF-α treatment in human chondrocytes in the presence or absence of p38 inhibitor SB203580, PKR inhibitor C16, p53 inhibitor Pifithrin-μ or AKT activator SC79 (*n* = 3; * *p* < 0.05 compared to control group. *p* < 0.05 compared to TNF-α group, *p* values were generated by ANOVA using the Tukey’s test).

PGC-1α is a transcriptional coactivator that regulates the genes associated with mitochondrial biogenesis and decreased expression of PGC-1α was observed in human OA chondrocytes [35]. PKR is a critical stress sensor and molecular mediator of mitochondria function, thereby modulating stress-induced cell death [36]. In order to investigate whether activation of PKR resulted in repression of PGC-1α, we examined its expression in chondrocytes after poly (I:C) treatment. Results from this investigation revealed that inhibition of PGC-1α was observed in response to poly (I:C) (Figure 4A,B). Our results demonstrated that the reduced expression of PGC-1α after TNF-α stimulation was reversed by activation of PKR/p38 MAPK/p53 pathway (Figure 4C,D). The expression of PGC-1α was not lessened by TNF-α in the presence of AKT activator SC79 also suggested that downregulation of PGC-1α required the reduced expression of AKT.

### 3.5. TNF-α-Induced Apoptosis in Chondrocytes Is Mediated by the PKR/p38 MAPK/p53/AKT/PGC-1α Pathway

**Figure 5:**
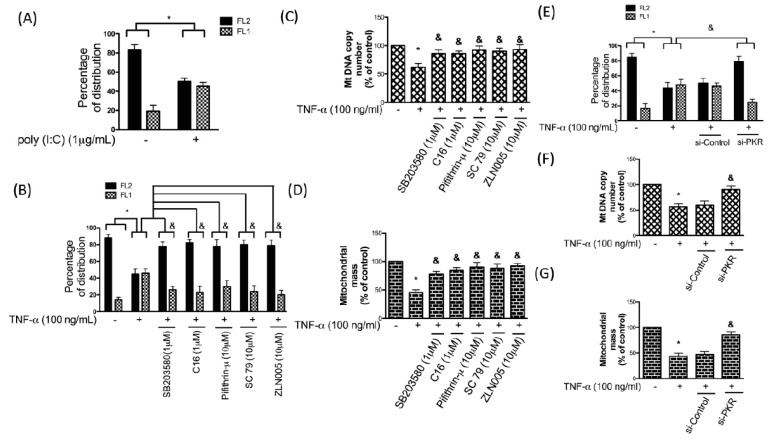
Percentage of cells expressing JC-1 aggregates (red fluorescence; FL2) and JC-1 monomers (green fluorescence; FL1) were assessed using flow cytometry with or without poly I:C treatment. (**A**,**B**) Percentage of cells expressing FL2 and FL1, (**C**) mitochondrial DNA copy number, and (**D**) mitochondrial mass were examined after administration of TNF-α with p38 inhibitor SB203580, PKR inhibitor C16, p53 inhibitor Pifithrin-μ, AKT activator SC79, or PGC-1α stimulator ZLN005. (**E**) Percentage of cells expressing FL2 and FL1, (**F**) mitochondrial DNA copy number, and (**G**) mitochondrial mass were evaluated in TNF-α-stimulated chondrocytes in the presence of small-interfering (si)-PKR or si-Control. *n* = 3; * *p* < 0.05 compared to control group. *p* < 0.05 compared to TNF-α group, *p* values were generated by ANOVA using the Tukey’s test.

Subsequently, we used the membrane-permeant JC-1 dye to measure apoptosis using flow cytometry. As shown in Figure 5A, we observed a higher percentage of cells expressing JC-1 red fluorescence (FL2) in healthy cells, whereas cells with poly (I:C) treatment displayed the reduced percentage of JC-1 red fluorescence (FL2) and the increased green fluorescence (FL1). Additionally, we found that TNF-α treatment caused the same indication of apoptosis and inhibition of PKR/p38 MAPK/p53 pathway prevented this phenomenon (Figure 5B). Moreover, the application of AKT activator SC79 or PGC-1α stimulator ZLN005 avoided the TNF-α-induced apoptosis (Figure 5B), suggesting that downregulation of AKT and PGC-1α also participated in this change.

Mitochondria are associated with various biological activities, including free oxygen radical generation and cell apoptosis. It has been shown that mitochondrial mass and mitochondria DNA (mtDNA) altered in response to oxidative stress [37]. We showed that TNF-α decreased the mtDNA DNA copy number (Figure 5C) and mitochondrial mass (Figure 5D) in chondrocytes, while suppression of the PKR/p38 MAPK/p53 pathway reversed them (Figure 5C,D). Likewise, administration of AKT activator SC79 or PGC-1α stimulator ZLN005 reverted the TNF-α-induced mitochondria dysfunction (Figure 5C,D). To further confirm the significance of PKR in this regulation, we used si-PKR and showed that the TNF-α-induced apoptosis, reduced mtDNA DNA copy number and mitochondrial mass were all abrogated after knockdown of PKR (Figure 5E–G). The knockdown efficiency of PKR and inhibition efficiency of inhibitors had been confirmed in our previous study [16].

### 3.6. PKR is Associated with the TNF-α-Induced Oxidative Stress in Chondrocytes

**Figure 6:**
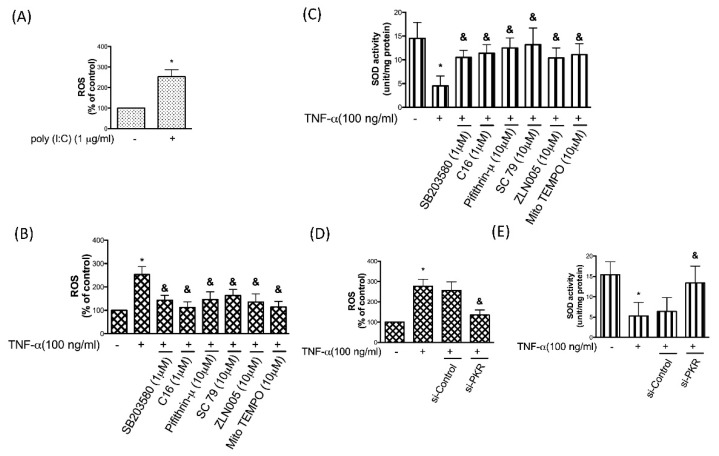
ROS production was elevated in chondrocytes treated with poly (I:C). (**A**,**B**) ROS generation and (**C**) SOD activity were determined in the TNF-α-stimulated chondrocytes with p38 inhibitor SB203580, PKR inhibitor C16, p53 inhibitor Pifithrin-μ, AKT activator SC79, PGC-1α stimulator ZLN005, or a specific scavenger of mitochondrial superoxide Mito TEMPO. (**D**) TNF-α-induced ROS production and (**E**) TNF-α-inhibited ROS in chondrocytes were interfered by si-PKR. *n* = 3; * *p* < 0.05 compared to control group. *p* < 0.05 compared to TNF-α group, *p* values were generated by ANOVA using the Tukey’s test.

Next, we showed that elevation of PKR resulted in higher ROS (Figure 6A), and administration of TNF-α also generated the increased ROS (Figure 6B) and decreased activity of the anti-oxidant SOD (Figure 6C). Our results supported that the upregulation of ROS and downregulation of SOD activity were regulated by the PKR/p38 MAPK/p53/AKT/PGC-1α pathway (Figure 6B,C). Moreover, we demonstrated that the TNF-α-stimulated ROS generation and SOD suppression were abolished in the presence of MitoTEMPO, a specific scavenger of mitochondrial superoxide (Figure 6B,C) as well as in the PKR-knockdown chondrocytes (Figure 6D,E).

### 3.7. TNF-α-Induced Apoptosis in Chondrocytes Is Due to the Accumulation of Oxidative Stress via PKR/p38 MAPK/p53/AKT/PGC-1α Signaling

**Figure 7:**
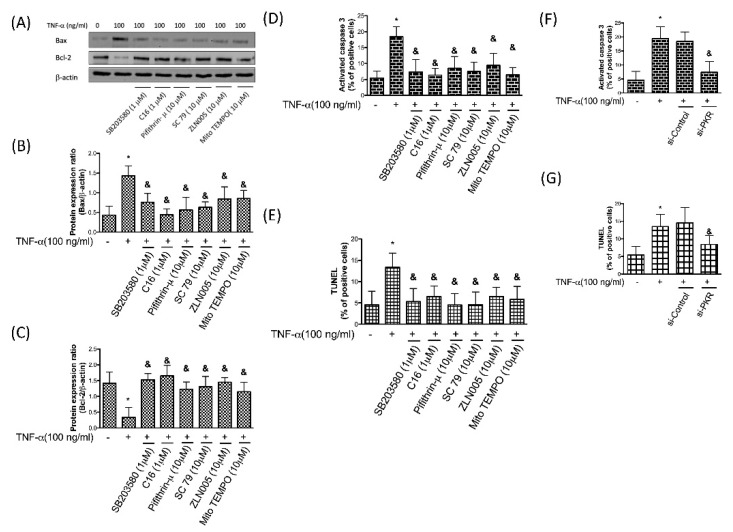
Protein expression (**A**) and ratio of Bax and Bcl2 (**B**,**C**) after TNF-α treatment in human chondrocytes in the presence of p38 inhibitor SB203580, PKR inhibitor C16, p53 inhibitor Pifithrin-μ, AKT activator SC79, PGC-1α stimulator ZLN005, or superoxide scavenger Mito TEMPO. Percentages of TNF-α-stimulated caspase-3 activation (**D**,**F**) and TUNEL-positive cells (**E**,**G**) were evaluated in response to p38 inhibitor SB203580, PKR inhibitor C16, p53 inhibitor Pifithrin-μ, AKT activator SC79, PGC-1α stimulator ZLN005, or superoxide scavenger Mito TEMPO. Percentages of TNF-α-stimulated caspase-3 activation (**C**) and TUNEL-positive cells (**D**) in normal, si-Control or si-PKR chondrocytes. *n* = 3; * *p* < 0.05 compared to control group. *p* < 0.05 compared to TNF-α group, *p* values were generated by ANOVA using the Tukey’s test.

The mitochondrial-mediated apoptosis is regulated by the Bcl-2 family of anti-apoptotic (such as Bcl-2) and pro-apoptotic proteins (such as Bax) [38]. It has been known that an increased Bax/Bcl-2 ratio upregulates caspase-3, which in turn increases apoptosis [39]. We showed that TNF-α increased the pro-apoptotic Bax and reduced the anti-apoptotic Bcl-2 in chondrocytes, whereas repression of PKR/p38 MAPK/p53/AKT/PGC-1α signaling or reduction of oxidative stress prevented this alteration (Figure 7A–C). Consistent with this finding, we showed that the TNF-α-induced caspase-3 activation (Figure 7D) and TUNEL-positive apoptotic cells (Figure 7E) were blocked by inhibition of the same signaling pathway. Most importantly, we proved that knockdown of PKR prevented the TNF-α-induced apoptosis in chondrocytes (Figure 7F,G), which may be a potential therapeutic target.

## 4. Discussion

**Figure 8:**
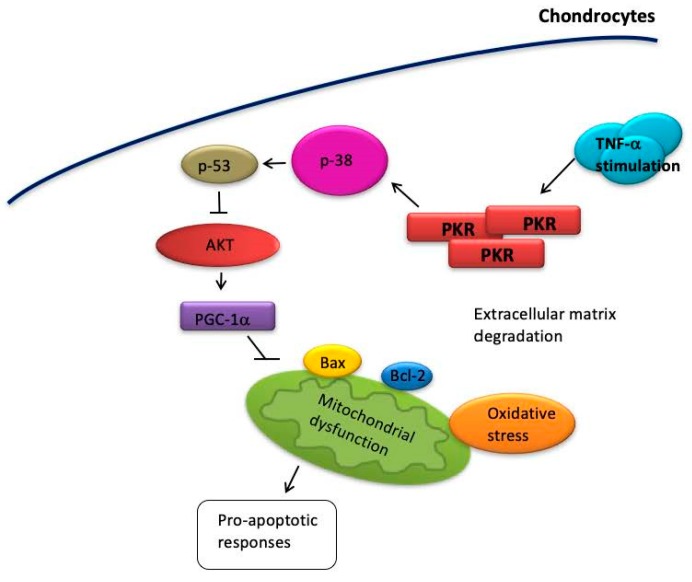
Phosphorylation of p38 MAPK by PKR activation elevates the expression of p53, leading to downregulation of AKT and the following suppression of PGC-1α. Subsequently, aberrant mitochondrial biogenesis (decreased the mitochondrial DNA copy number and mass) and enhanced oxidative stress (higher ROS and lower SOD activity) correlates with higher Bax and lower Bcl-2 expression with increased caspase-3 and TUNEL-positive cells, which may contribute to the pathogenesis of OA.

Chondrocyte apoptosis has been considered as an important step in the pathogenesis of cartilage destruction [8,9,10]. Apart from IL-1β, TNF-α is another pro-inflammatory cytokine produced by chondrocytes that have been shown to result in deterioration of OA [40] and apoptosis of chondrocytes [13]. Over the past decades, numerous studies have focused on the activation of MMPs in chondrocytes by TNF-α [16,41,42], however, its precise role and associated mechanism in chondrocyte apoptosis have not been fully elucidated. In the current report, we demonstrated that TNF-α-induced apoptosis in chondrocytes was due to the accumulation of oxidative stress via PKR/p38 MAPK/p53/AKT/PGC-1α signaling pathway (Figure 8). It has been found that OA chondrocytes had decreased levels of phosphorylation of AMPKα and expression of SIRT1 and PGC-1α compared to normal donor chondrocytes. The decreased protein expression of the mitochondrial biogenesis mediators and reduced mitochondrial DNA content and mitochondrial mass were also reported [35]. Our results demonstrated consistent findings of mitochondrial dysfunction with reduced PGC-1α, decreased mitochondrial DNA copy number and mass in chondrocytes after TNF-α stimulation. In accordance with our findings, one of the previous studies has demonstrated that reduction of oxidative stress in chondrocytes counteracted the upregulated expression of apoptotic cascade factors caused by TNF-α [43], indicating oxidative stress as a key part to the TNF-α-induced apoptosis. The antioxidant deficiency has been revealed in OA chondrocytes with lower SOD activity and an increase of intracellular ROS production [44], and we proved that these changes might occur as a result of TNF-α activation. In addition, downregulation of SOD has been considered as relevant to mitochondrial dysfunction in OA [45], which is often associated with apoptosis. In this study, we linked the reduced mitochondrial biogenesis capacity in TNF-α-stimulated chondrocytes with increased oxidative stress and the subsequent apoptosis, evidenced by the markedly elevated Bcl-2 and caspase-3 expression.

Secondly, we demonstrated that the activation of PKR was implicated in TNF-α-induced apoptosis in chondrocytes. Apart from serving as a mediator of the increased MMPs secretion that leads to cartilage degradation [16,18,46], various reports have suggested that PKR functions as a general transducer of the apoptotic response [19,47,48]. The involvement of PKR has been revealed in TNFα-induced apoptosis in NIH3T3 cells, and they showed that overexpression of PKR is sufficient to induce apoptosis [20]. Consistent with this result, we showed that the TNF-α-activated caspase 3 and % of TUNEL positive cells were downregulated in si-PKR chondrocytes. Moreover, we unveiled the immediate substrates downstream of PKR that lead to apoptosis. We showed that PKR activation triggered the p38 MAPK/p53/AKT pathway, leading to reduction of PGC-1α and the following aberrant mitochondrial biogenesis. Another MAPK ERK1/2 has been shown to mediate caspase-3-dependent apoptosis induced by TNF-α in human chondrocytes as well [49]. Several studies have shown that p38 MAPK-mediated p53 phosphorylation represents a critical step of apoptosis [32,33] and p53 induction was a downstream event of TNF-α-induced up-regulation of PKR followed by apoptosis in U937 cells [50]. In fact, PKR can also function as a target of p53. It has been reported that activation of p53 induced by DNA damage facilitated cell apoptosis via PKR [51].

Over the past decades, the role of PKR in OA development has been investigated in various studies. It has been demonstrated that phosphorylation of PKR occurred in cyclic loaded porcine articular cartilage explants and inhibition of PKR modestly reversed the global suppression of protein synthesis caused by cyclic loading, indicating that PKR was mediated the loading-induced translational arrest [52]. Furthermore, PKR has been indicated to mediate the TNF-α- and IL-1 induced activation of MMP-2 and -9 [18,46]. Moreover, the PKR inhibitor reduced the accumulation of COX-2 and PGE2 in IL-1α-activated cartilage [17]. In our previous study, we revealed that PKR activation resulted in oxidative stress accumulation and exaggerated inflammatory response with increased COX-2 and IL-8 via ERK/NF-κB pathway. We showed that activated ERK pathway impeded the inhibition of MMP-13 by PPAR-γ, and MMP-13 is the key to the pathogenesis of cartilage degradation [16]. In the current study, we focused on the detailed mechanism underlying the PKR-mediated oxidative stress. We showed that PKR-mediated oxidative stress and damages led to p53 phosphorylation via p38 MAPK. The following suppression of Akt and PGC-1α ultimately promoted the commitment of chondrocytes to apoptosis with an increased Bax/Bcl-2 ratio and caspase-3 along with mitochondrial dysfunction. Both of these two studies supported that PKR plays a critical role in regulation cartilage and chondrocytes dysfunction. PKR has also been shown to play a role in the differentiation of chondrocytes through the modulation of STAT1 and Sox-9 expression [53]. Altogether, these findings suggested that PKR might be critical to the pathogenesis of OA as it participated in the degradation of cartilages via MMPs production and apoptosis induction as a result of mechanical loading or inflammatory response.

There is a limitation in this study, we proved that PKR causes chondrocyte apoptosis and oxidative injuries via modulation of p38 MAPK. However, p38 MAPK is activated through many modulators, such as MKK6 or FAK. In this study, we did not answer if MKK6 or FAK is involved this PKR-upregulated p38 MAPK. This issue will be our future direction for further study.

## 5. Conclusions

In summary, the present study demonstrated that PKR mediated the TNF-α-induced oxidative stress in human chondrocytes via p53 phosphorylation by p38 MAPK, leading to repression of AKT and PGC-1α. We showed that this alteration promoted the commitment of chondrocytes to apoptosis with an increased Bax/Bcl-2 ratio and caspase-3 expression following mitochondrial dysfunction (Figure 8). Hence, suppression or inhibition of PKR may be a promising therapeutic approach to diminish the cartilage damage caused by inflammation.

## Abbreviations

OA: osteoarthritis; PKR, protein kinase R; eIF-2α, eukaryotic initiation factor 2; MMP, matrix metalloproteinases; COX, cyclooxygenase; NAO, N-nonyl acridine orange; poly (I:C), polyinosinic-polycytidylic acid.
